# The Prognostic Value of Natriuretic Peptides in Stable Patients with Suspected Acute Myocarditis: A Retrospective Study

**DOI:** 10.3390/jcm11092472

**Published:** 2022-04-28

**Authors:** Gassan Moady, Shahar Perlmutter, Shaul Atar

**Affiliations:** 1 Department of Cardiology, Galilee Medical Center, 1 Ben Tzvi Blvd, Nahariya 2210001, Israel; shaula@gmc.gov.il; 2 Azrieli Faculty of Medicine, Bar Ilan University, Safed 5290002, Israel; shaharperlmutter@gmail.com

**Keywords:** myocarditis, ventricular function, natriuretic peptide, risk stratification

## Abstract

Risk stratification in acute myocarditis is based on the clinical signs of heart failure, the degree of cardiac dysfunction, and the findings in cardiac magnetic resonance (CMR). The aim of the current study is to examine the prognostic yield of the natriuretic peptide N-terminal-pro hormone Brain Natriuretic Peptide (NT-proBNP) and C-reactive protein (CRP) in acute myocarditis among patients with preserved/mildly reduced left ventricular ejection fraction (LVEF). We retrospectively analyzed 59 patients (median age 28 years, 76% males) with ICD-9 discharge diagnosis of acute myocarditis. Basic characteristics, echocardiographic, and laboratory parameters were obtained from computerized files. The median length of stay was 3, (IQR 2–5) days, and the median LVEF was 48% (IQR, 54–62%). High levels of NT-proBNP and CRP were associated with increased length of stay (r = 0.57, *p* < 0.001; r = 0.4 *p* = 0.001, respectively), while troponin level was not (r = 0.068, *p* = 0.61). During the index hospitalization, complications occurred in 14 (23.7%) patients. High NT-proBNP and CRP levels were associated with complications (*p* < 0.001, and *p* = 0.001, respectively), while troponin level was not (*p* = 0.452). In conclusion, routine measurement of NT-proBNP and CRP are preferred over troponin for risk stratification in hemodynamically stable myocarditis.

## 1. Introduction

**Figure 1:**
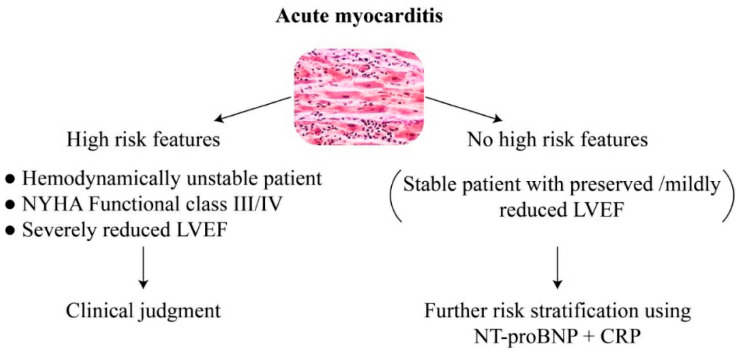
Simple risk stratification in acute myocarditis using echocardiography, clinical parameters, natriuretic peptide, and CRP. NYHA New: York Heart Association; LVEF: Left Ventricular Ejection Fraction; NT-proBNP: N-terminal-pro hormone Brain Natriuretic Peptide; CRP: C-reactive protein.

Acute myocarditis is an inflammatory disease of the myocardium, typically seen following a viral disease in young adults, but may occur at any age. The global prevalence is about 22 of 100,000 patients annually [1,2,3], but the true incidence is difficult to estimate since the diagnosis is often missed due to non-specific symptoms of the disease [4]. The disease has a large spectrum of clinical presentation, ranging from mild non-specific symptoms to fulminant disease with acute heart failure and potential subsequent dilated cardiomyopathy, or sudden cardiac death (SCD) [5,6,7,8]. The diagnosis is based on clinical symptoms, laboratory tests, ECG changes, and cardiac imaging. Endomyocardial biopsy (EBM) was the gold standard for the diagnosis of myocarditis using immunohistological analyses and molecular biological detection of the microbial gene in the myocardium [9]. However, the restricted availability of EMB, the potential complications of this invasive procedure, and its low sensitivity in patchy diseases have led to its gradual replacement by CMR. The diagnosis of myocarditis by CMR was based traditionally on the Lake-Louise criteria, using T2 weighted ratio, early gadolinium enhancement, and late gadolinium enhancement [10]. Recently, novel mapping techniques using T1, T2 and extracellular volume mapping demonstrated additional diagnostic information in the acute and chronic phases of myocarditis [11,12]. Cardiac biomarkers are largely used for diagnostic and prognostic purpose in various cardiovascular conditions. Troponin is a cardiac marker that is typically elevated in conditions with myocardial injury, such as myocardial infraction and myocarditis. By definition, acute myocarditis is accompanied by elevation in myocardial injury biomarkers, with troponin as the most commonly used one, although normal troponin level does not exclude myocarditis [1,13]. Similar to other inflammatory diseases, acute myocarditis is associated with increased levels of CRP during the acute phase [1]. In addition, Nt-proBNP, a marker of wall stretch that may be elevated in several conditions such as heart failure, acute coronary syndrome, and pulmonary embolism [14,15,16,17], may also play a role in myocarditis. We hypothesized that NT-proBNP and CRP may predict the severity of the disease in acute myocarditis among patients with preserved or mildly reduced LVEF better than troponin. Our hypothesis is summarized in Figure 1.

In unstable patient or those with acute heart failure, biomarkers are less relevant for risk assessment, and patients should be treated in accordance with the guidelines of heart failure and the common practice. In patients with preserved or mildly reduced LVEF (40–50%), the use of NT-proBNP and CRP may be preferred over troponin for risk stratification.

NYHA New York Heart Association; LVEF left ventricular ejection fraction, NT-proBNP N-terminal-pro hormone Brain Natriuretic Peptide; CRP C-reactive protein.

## 2. Materials and Methods

### 2.1. Study Population

A total of 59 patients with ICD-9 diagnosis of myocarditis at discharge were included in the final analysis of this retrospective study. All patients were hospitalized in the cardiology department in the Galilee Medical Center, Nahariya, Israel.

We included patients with discharge diagnosis of acute myocarditis and with available laboratory data (including NT-proBNP). One patient with unstable hemodynamic and cardiogenic shock who needed mechanical circulatory support, and one patient with LVEF < 40% were excluded from the final analysis. In the majority of the patients, the diagnosis of myocarditis was highly suspected based on clinical presentation of chest pain following viral disease, fever, palpitations, elevated troponin level, ECG changes, and echocardiographic findings. Patients with high probability of acute coronary syndrome based on risk profile, had a coronary angiography performed, and for selected patients with questionable diagnosis, CMR was also performed. All patients were in stable respiratory and hemodynamic condition and treated in the cardiology department. Patients received antipyretic and analgesia for symptom relieve when needed. During the hospital stay, they were monitored for arrhythmia, and had ECG and laboratory tests performed every day and whenever indicated according to clinical judgment. The following ECG changes were recorded pathological: ST-segment depression 80 ms following the J-point and T-wave inversion at the nadir. Atrial and ventricular arrhythmia were also captured. Echocardiography was performed by the same cardiologist.

### 2.2. Echocardiographic Data

All patients underwent echocardiography using Philips Epiq-7 machine with EPIQ X8-2t transducer (Phillips, Andover, MA, USA). Parameters were obtained and calculated by the same cardiologist expert in echocardiography who was blinded for the study design. LVEF was calculated by the Simpson’s biplane method in the apical four-chamber and two-chamber views using the formula: Ejection fraction (EF) = Left ventricular end diastolic volume-Left ventricular end systolic volume)/Left ventricular end diastolic volume X100. Left ventricular end diastolic diameter was measured in the parasternal long-axis view. The E/e’ was calculated by the average of septal and lateral E/e’. Left atrial volume index (LAVI) was measured at the end of ventricular systole using the formula: LAVI = Left atrial volume/Body surface area.

### 2.3. Laboratory Data

High sensitivity troponin I (Hs-TnI) level was measured using ARCHITECT c-TnI assay, Abbott. Cut-off values for abnormal Hs-TnI levels were above 20 ng/L and 30 ng/L for men and women, respectively. NT-proBNP is expressed in pg/mL and was determined using electrochemiluminescence Elecsys immunoassay (Roche, diagnostics), with abnormal levels above 125 pg/mL. Other laboratory values included C-reactive protein, Hemoglobin, white blood cells, and kidney function. For each patient, we used maximal available values of troponin, WBC, CRP and troponin during the index hospitalization.

### 2.4. Statistical Analysis

Qualitative parameters are expressed in prevalence and percentage, while Quantitative parameters in median with interquartile range (IQR). Spearman’s test was used to examine the correlation between laboratory values and length of stay. Wilcoxon rank sum test was used to test the correlation between different parameters and the occurrence of complications. Linear multivariant regression analysis was used to predict length of stay by various parameters. With two-sided alpha of 5% considered significant, a total of 49 patients were needed to achieve 80% statistical power. Statistical analysis was performed using IBM SPSS statistics, 27. The study was approved by the local ethical committee in Galilee Medical Center.

## 3. Results

The median age of the study population was 28 years with 76.3% males. The majority have no background coronary artery disease. The basic characteristics of the patients are given in Table 1.

The echocardiographic data are shown in Table 2.

Among the 59 patients, 11 (18.6%) had coronary angiography which revealed no coronary artery obstruction, and another 10 (16.9%) underwent CMR which confirmed the diagnosis of myocarditis based on tissue mapping techniques. During the index hospitalization, 14 (23.7%) patients experienced complications including atrial fibrillation (5.1%), ventricular tachycardia (10.2%), syncope (3.4%), and pulmonary congestion (5.1%). The length of stay was driven by the persistence of symptoms and the presence of complications. The mortality rate was 0%, and no patients required mechanical ventilation or circulatory support. High levels of NT-proBNP and CRP were associated with increased length of stay (r = 0.57, *p* < 0.001; r = 0.4, *p* = 0.001, respectively), while troponin level was not (r = 0.068, *p* = 0.61). Eighteen (30.5%) patients had NT-proBNP within normal limits; none of them had complications. Higher levels of NT-proBNP and CRP were correlated to the occurrence of complications during the index hospitalization (*p* < 0.001, and *p* = 0.001, respectively), while on the other hand, hs-TnI level was not (*p* = 0.452). On discharge, 55 (93.2%) patients had complete normal echocardiography exam and NT-proBNP within normal limits. Four patients with persistent elevated NT-proBNP and mildly reduced LVEF (LVEF 45–50%) were followed -up at our heart failure clinic and were putted on neurohormonal medications including ACE inhibitors and beta blockers with complete recovery and subsequent decrease in NT-proBNP levels during 1-years follow-up. After three years follow-up no adverse events or rehospitalizations were recorded based on the computerized files.

The laboratory tests, hemodynamic parameters, and outcomes are summarized in Table 3.

**Figure 2:**
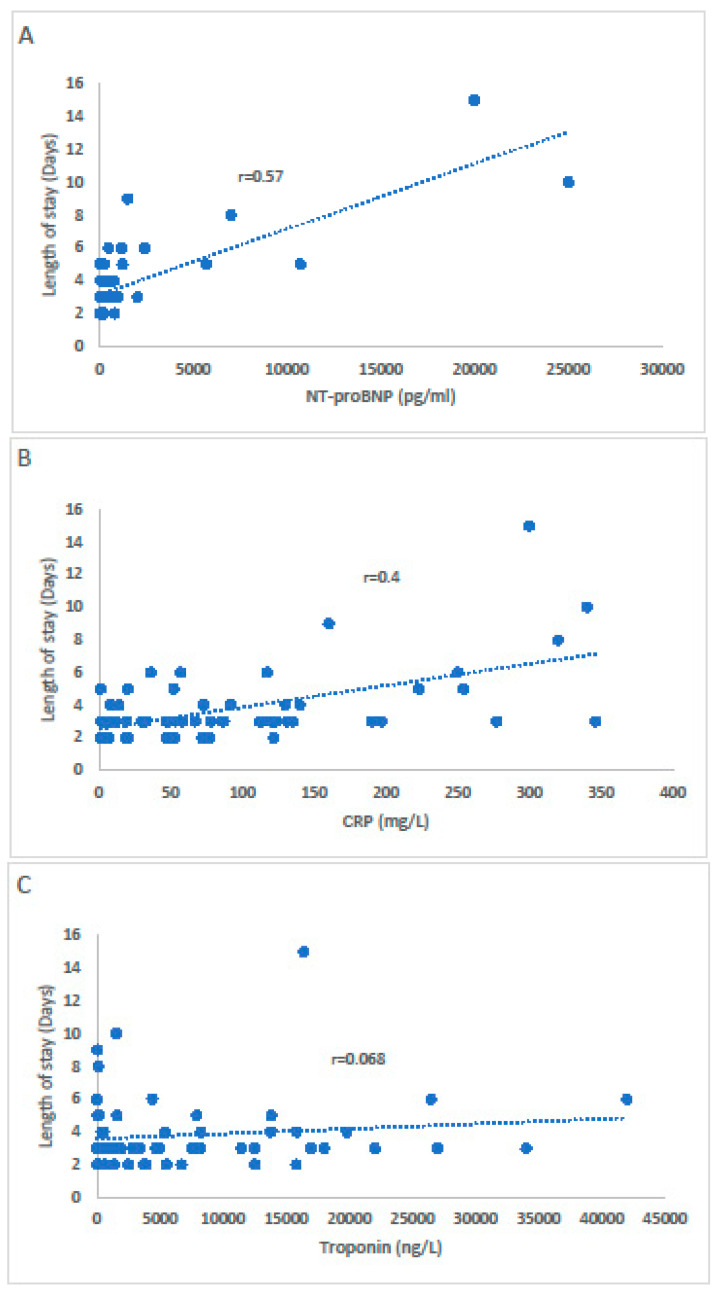
The correlation between NT-proBNP (**A**), CRP (**B**), and troponin (**C**) with the length of stay.

The correlation between the length of stay and the markers (NT-proBNP, CRP, and troponin) are shown in Figure 2A–C.

A statistically significant correlation was observed between NT-proBNP (A), CRP (B) and the length of stay, while there was no correlation with troponin level (C).

## 4. Discussion

Risk stratification in acute myocarditis is of paramount significance and should rely on clinical, laboratory, and imaging parameters. In one study, advanced New York Heart Association (NYHA) functional class (III/IV), reduced LVEF (<35%), and right ventricular ejection fraction ≤45% in patients with myocarditis were associated with SCD [18]. In another study, the most powerful predictors of mortality in severe myocarditis were advanced NYHA functional class, immunohistological evidence of infiltrate, and the lack of beta-blockers therapy [19]. Among the various imaging modalities, the amount of late gadolinium enhancement detected on CMR is considered an independent risk factor for adverse cardiovascular events in non-ischemic cardiomyopathy [20,21]. The use of CMR for diagnostic and prognostic purposes, however, may not be always available or indicated in stable patients particularly when the cardiac function is preserved. In the current study, we aimed to examine the prognostic value of different biomarkers including NT-proBNP and troponin in stable patients with preserved or mildly reduced LVEF and clinically suspected acute myocarditis. The routine use of NT-proBNP in acute myocarditis is simple, widely available, and may predict persistent symptoms (reflected by prolonged hospital stay) and complications better than troponin. Although cardiac troponin is an indicator of cardiac tissue injury, it was not correlated in our study population to increased risk of complications or prolonged symptoms, and therefore may not be reliable for risk assessment in this population. The Brain Natriuretic Peptide (BNP) is released following myocardial wall stretch [22,23]. In response to these pathological conditions, the ventricles produce preproBNP peptide, which is then cleaved into proBNP by removing the N-terminal 26 amino acid signal peptide. The molecule proBNP is then cleaved by the endopeptidase Curin/Furin into BNP and the inactive NT-proBNP [24,25]. Subsequently, high levels of NT-proBNP may be observed in various conditions, including heart failure, acute coronary syndrome, and pulmonary embolism [14,15,16,17]. The level of NT-proBNP in the serum correlates with the severity of the disease in the aforementioned conditions. In acute myocarditis, the edema caused by the inflammatory process in the myocardial tissue may increase the wall stress and induce the activation of the neurohormonal axis, leading finally to the release of BNP. Several cardiac biomarkers have been investigated for diagnosis and risk stratification in the setting of acute myocarditis [26,27,28]. In their study, Ukena et al. demonstrated that hs-TnT, but not Copeptin nor mid-regional pro-adrenomedullin (MR-proADM), is a predictive marker for biopsy-proven acute viral myocarditis, while NT-proBNP concentration above the 4th quartile was the only biomarker to be associated with cardiac death or the need for heart transplantation following acute viral myocarditis [28]. In another study, higher NT-proBNP level was associated with higher CRP, leukocytes, and neutrophile to lymphocyte ratio, indicating inflammation, however it was not correlated with LVEF or mortality [29]. Recently, a novel microRNA, specific for myocarditis, demonstrated an excellent accuracy in distinguishing acute myocarditis from acute myocardial infarction even after adjustment for age, sex, ejection fraction, and troponin level [30]. For risk stratification, the combination of highly elevated troponin and mild elevation of NT-proBNP was observed in patients with fulminant myocarditis [31]. Our results support previous data regarding the lack of correlation between high troponin and poor prognosis in myocarditis [32]. In one study involving pediatric patients with myocarditis, highly elevated troponin was not associated with ventricular dysfunction or poor outcomes [33]. In another CMR-based study, the level of myocardial damage detected by CMR during acute myocarditis was positively correlated to the level of CRP while troponin level was not [34]. Our data, along with previous studies support the prognostic value of CRP and NT-proBNP, but not troponin in the acute phase of myocarditis. This disproportion in biomarkers levels may be explained by the inability of the exhausted ventricles in fulminant myocarditis to promote neurohormonal activation [31]. This is consistent with the finding of relatively low NT-proBNP levels in some patients with end-stage heart failure, particularly those with higher body mass index [35]. Patients with fulminant myocarditis often present with advanced NYHA functional class secondary to massive myocardial necrosis and reduced LVEF, while in patients with mild disease, the dominant process is inflammation reflected by high CRP levels. The amount of wall stress in the myocardial tissue caused by the inflammation (reflected by elevated natriuretic peptide) may predict the severity of the disease in patients with preserved cardiac function. The limited use of CMR in some centers and the need for additional non-invasive test for rapid risk stratification make the use of NT-proBNP for this purpose attractive. The serial measurement of NT-proBNP and CRP is superior to troponin for risk stratification. Patients with high NT-proBNP levels are more likely to suffer from prolonged symptoms, and need special attention for potential complications. This biomarker should be considered for risk assessment along with echocardiography and clinical judgment.

## 5. Limitations

First, the diagnosis of myocarditis was not confirmed by EMB or CMR in all patients (CMR in 10 patients, and coronary artery disease was ruled out in 11 patients). However, clinically suspected myocarditis is an acceptable diagnosis in our study population based on the lower cardiovascular risk profile and the very suggestive scenario, making other diagnosis very unlikely. We can assume that after exclusion of other causes such as acute coronary syndrome, tachyarrhythmia, hypertensive crisis, and acute heart failure, elevation in high sensitivity troponin in the clinical context of viral infection and in patients with chest pain, is highly suggestive for acute myocarditis Moreover, our results support the prognostic value of NT-proBNP in patients with chest pain and elevated troponin regardless of the final diagnosis. Second, the small number of the patients mandates further validation for patients with larger spectrum of disease severity.

## 6. Conclusions

In patients with suspected acute myocarditis, the level of NT-proBNP and CRP were positively correlated with the severity of the disease, while troponin was not. Routine measurement of NT-proBNP and CRP are preferred over troponin for risk stratification in the cases of hemodynamically stable myocarditis.

## Figures and Tables

**Table 1:** Basic characteristics of the study population.

Age (Years)	28 (20–43)
Male	45 (76.3%)
BMI (Kg/m^2^)	26.1 (18.1–27)
Current smoker	17 (28.8%)
Diabetes mellitus	6 (10.2%)
Hyperlipidemia	12 (20.3%)
Hypertension	6 (10.2%)
Coronary artery disease	1 (1.7%)
Family history of coronary artery disease	15 (25.4%)
Chronic kidney disease	3 (5.1%)
Chronic obstructive pulmonary disease	2 (3.4%)
Chronic medications	
ACE inhibitors/ARBs	5 (8.5%)
Beta blockers	6 (10.2%)
Statins	10 (16.9%)
Aspirin	3 (5.1%)
Eltroxin	5 (8.5%)
Clinical presentation	
Chest pain	52 (88.1%)
Dyspnea	12 (20.3%)
Palpitations	7 (11.9%)
Viral prodrome	50 (84.9%)

ACE: Angiotensin-converting enzyme; ARBs: Angiotensin II receptor blockers; BMI: body mass index.

**Table 2:** Echocardiographic parameters.

LVEF (%)	48% (54–62)
LVEDD (mm)	47 (44–50)
LAVI (mL/m^2^)	23 (20–26)
E/e’	8.5 (7.2–9.3)
Pericardial effusion	12 (20.3%)
Mitral valve regurgitation	3 (5.1%)
Tricuspid valve regurgitation	4 (6.8%)
Estimated SPAP (mmHg)	22 (16–28)
TAPSE (cm)	1.8 (1.7–2.0)

LVEDD: Left ventricular end diastolic diameter; LVEF: Left ventricular ejection fraction; LAVI: Left atrial volume index; SPAP: Systolic pulmonary artery pressure; TAPSE: Tricuspid Annular Plane Systolic Excursion. Mitral valve regurgitation was reported when it was more than minimal, and pericardial effusion when it is more than minimal in the subcostal or apical-four chamber views.

**Table 3:** Basic hemodynamic, laboratory and outcomes.

Pulse (BPM)	76 (70–88)
SBP (mmHg)	116 (110–125)
DBP (mmHg)	73 (64–80)
Spo_2_ (%)	96 (94–98)
Respiratory rate	14 (12–18)
Temp (°C)	37 (36–37.8)
ECG changes	35 (59.3%)
NT-proBNP pg/mL	300 (59–830)
Hs-TnI (ng/L)	3435 (373–12,500)
CRP (mg/L)	67 (20–130)
WBC (×10^9^/L)	10 (8–13.7)
Hemoglobin (g/dl)	14 (13–15)
Creatinine (mg/dl)	0.83 (0.7–0.99)
eGFR (CKD-EPI) (mL/min/1.73 m^2^)	117 (98–123)
Potassium (mmol/L)	4.0 (3.7–5.1)
Sodium (mEq/L)	139 (130–138)
Use of analgesia	50 (84.7%)
Invasive coronary angiography	11 (18.6%)
Cardiac MRI	10 (16.9%)
Complications	14 (23.7%)
Atrial fibrillation	3 (5.1%)
Ventricular tachycardia	6 (10.2%)
Syncope	2 (3.4%)
Pulmonary congestion	3 (5.1%)
Length of stay (days)	3 (2–5)

SBP: systolic blood pressure; DBP: diastolic blood pressure; ECG: electrocardiogram; NT-proBNP: N-terminal-pro hormone Brain Natriuretic Peptide; Hs-TnI: high-sensitivity troponin I; CRP: C-reactive protein; WBC: white blood cells; eGFR: estimated glomerular filtration rate; MRI: magnetic resonance imaging.
